# Psychometric properities of the Polish version of clinical learning environment inventory

**DOI:** 10.1186/s12912-021-00649-5

**Published:** 2021-07-08

**Authors:** Iwona Bodys-Cupak

**Affiliations:** Department of Nursing Fundamentals, Jagiellonian University Medical College, Faculty of Health Sciences, Institute of Nursing and Midwifery, 12 Michałowskiego Str., 31-126 Krakow, Poland

**Keywords:** Clinical learning experience, Nursing education, Nursing students, Psychometrics, Student satisfaction

## Abstract

**Background:**

Clinical experience is a crucial activity for nursing students. The way students` perceive clinical placement exerts an immense influence on the learning process. This study aims to test the psychometric properties of a 19-item version of the Clinical Learning Environment Inventory under Polish clinical conditions.

**Method:**

For this study, Discriminant validity and Cronbach’s alpha reliabilities were computed. In order to measure content validity, the criterion validity Generalized Self Efficacy Scale and the Life Orientation Test - Revised were used.

**Results:**

Cronbach’s Alpha for the Clinical Facilitator Support of Learning Scale and the Satisfaction with Clinical Placement scale is 0.949 and 0.901, respectively. The Spearman’s rank correlation coefficient indicates the existence of a positive correlation between the students’ satisfaction with clinical placement and their [overall] life optimism. Age correlates negatively with perceived teacher support and positively with satisfaction with clinical placement. The sense of self-efficacy correlates negatively with their satisfaction with clinical placement. Clinical Learning Environment Inventory − 19 could be a useful tool to evaluate the quality of the clinical learning process in Polish conditions.

## Background

Clinical placement is an irreplaceable learning environment, very not similar to the university classroom, and this experience is, therefore, a crucial activity in nursing instruction. The clinical environment provides an optimal place to learn from role-models and to practice nursing skills. It follows that the way students` perceive clinical placement exerts an immense influence on the learning process. The support of an experienced clinical facilitator and the diverse learning opportunities available in the clinical setting have been found as significant determinants of the level of student satisfaction from their clinical learning experience [1–3]. Students acquire skills most successfully in clinical environments that allow for their participation in care provision and enable them to work alongside healthcare staff supporting their learning and enhancing their motivation [4]. A constructive clinical learning atmosphere intensifies learning and proliferates the acquisition of necessary skills and information [5, 6].

Both the development of clinical skills and hands-on practice in clinical settings also enable professional socialisation [7]. It has been demonstrated that the way students perceive their clinical learning setting affects their overall success as well as their learning outcomes [8].

Nursing students` positive perceptions of the clinical learning environment contribute to a better level of self-assessed competence and satisfaction with the clinical experience, leding to lower turnover intentions [9, 10]. Nursing student’s perspective in the clinical placement maximizies the students` willingness to learn [11].

The Clinical learning environment is understood as the work setting in a clinical healthcare facility, in which nursing students undertake their clinical placements to complete their medical education [12]. The training of nursing students takes place in a variety of environments and health care facilities, wherein students play a double role as both learners and patient carers. Clinical education occurs in a context that is more conducive to providing medical services than to learning. Therefore, any lack of supervision over this environment may affect the value of nursing education [13]. Quality assessment of the clinical learning environment is an essential means to guarantee the clinical know-how of future nursing specialists, which serves to heighten patient welfare.

The Clinical Learning Environment Inventory is among some of the most common surveys used to evaluate students’ perception of the clinical learning setting [14, 15]. This tool is used to rate the actual clinical learning setting as well as the students’ expectations in such an environment.

Therefore, the modified Clinical Learning Environment Inventory - short version CLEI-19 [16], originating from the Clinical Learning Environment Inventory [17], was selected for this study.

The CLEI-19 survey is useful in examining two factors of clinical experience: Clinical Facilitator Support of Learning and Satisfaction with Clinical Placement. These aspects of support are crucial because they impact the knowledge acquired during classes and the development of practical skills, two elements essential for nursing students if they are to become fully competent health-care professionals.

This study aimed to examine the psychometric properties of a 19-item version of the CLEI in Polish clinical conditions.

### Design

The design of the study was a cross-sectional, descriptive survey.

### Characteristic of the CLEI-19

The CLEI-19 survey (Abbreviated Clinical Learning Environment Inventory) by Salamonson et al. evaluates teacher support of learning as perceived by the students as well as the students’ satisfaction with their placement. The inventory contains 19 statements, with 12 relating to Clinical Facilitator Support of Learning and seven relating to Satisfaction with Clinical Placement. In each statement, the respondent decides how much it is consistent with their feelings by selecting an answer from 1 (Strongly disagree) to 5 (Strongly agree) on a 5-point Likert scale. Omitted or invalid items are scored 3 [17]. Out of 19 statements in the CLEI-19, ten are positive and nine are negative, therefore, about half of the items are reverse-scored. CLEI-19 scores are between 19 and 95, with higher values demonstrating greater satisfaction with the clinical learning environment [16]. The facilitator support scale consists of 12 questions scored from 1 to 5, so its value ranges between 12 and 60 points. The satisfaction scale consists of 7 questions scored from 1 to 5, so its value ranges between 7 and 35 points. Higher scores represents more significant perceived support and greater satisfaction. Chan and Salamonson et al. demonstrated the reliability of their tools with Cronbach’s alpha coefficients of 0.73–0.84, and 0.93 for Chan’s CLEI and Salamonson et al. ‘s CLEI-19, respectively [16, 17].

### Procedure

The study covered nursing students in the first, second, and third year of their first-degree programme at the Faculty of Health Sciences of the Jagiellonian University Medical College. The study was conducted in 2018 (test, *N* = 307) and 2019 (retest, *N* = 126). The students were included in the study if they were first-degree full-time nursing students, had completed their practical classes, and had given their informed verbal consent to participate in the study. The students were excluded if they left one or more study semesters or were transferred from other nursing faculties during data collection (research duration), or after foreign internship or dean’s leave. The students were informed of the confidentiality and anonymity of the survey, that the contribution was voluntary, and that they might withdraw from participating in the study at any time. The differences in the sample size between the first (test) and second (re-test) test resulted from several factors. The study conducted in 2019 did not include 69 students who completed first-degree studies. Forty-seven students did not continue their studies in the second year because they either quit after the first year or failed the required exams or applied for a dean’s leave. As a result, 186 questionnaires were distributed to students who participated in the first study, identifying them according to the code assigned at the beginning of the study. One hundred sixty-one completed questionnaires were received, of which only 126 were correctly completed and finally incorporated into the analysis.

The questionnaires received from the respondents were scored and verified individually in terms of completeness, and the data was encoded, entered into the database, and processed using version 3.6.1. of the R software Core Team [18].

The author of the present study had obtained original questionnaire and prior consent from the authors of the original CLEI-19 survey to use the tool in the study. Psychometric testing of the original abbreviated Clinical Learning Environment Inventory survey used in the study has previously been published [16]. The independent translations from English into Polish were prepared by an English philologist experienced in translating scientific papers, two practising nurses, and an independent academic specialising in the field of health sciences. The analysis and comparison of all the translations demonstrated their close similarity. The Polish text of the questionnaire thus obtained was then back-translated by two independent translators. The comparison of the original version of the questionnaire with the back translation confirmed a high degree of compliance of all translations in terms of word choice and content. To maintain the facade equivalence of the questionnaire, the Polish language version had the same graphic layout. The method of conducting the study was consistent with the proposals of Salamonson et al. [16] during the validation of the original version of the questionnaire.

To maintain the fidelity of the reconstructed text, the reliability of the Polish language version was checked in the same way as when validating the original version. Standard recommendations were adhered to when verifying the reliability of the CLEI-19 questionnaire [19]. In the validation procedure, internal consistency was assessed to verify the test reliability, including the analysis of statistical properties of test items and the analysis of the relationship of test items with the overall test score (discriminating power). The approach utilised during reliability evaluation involved the analysis of the internal consistency of each subscale with the formula proposed by Cronbach. To assess the interscale correlations of individual statements, their discriminating powers were determined and a correlation value of at least 0.20 was adopted as the condition for sufficient differentiation [20].

For two groups, the values of quantitative variables were compared using the Mann–Whitney U test. The comparison of quantitative variable values in three or more groups was performed using the Kruskal-Wallis test. Having detected statistically significant differences, a posthoc analysis was performed with Dunn’s test to identify groups that showed any statistically significant differences. Correlations between quantitative variables were analysed using the Spearman’s rank correlation coefficient. The strength of dependence was interpreted according to the following formula: |r| ≥ 0.9 – very strong correlation; 0.7 ≤ |r| <  0.9 – strong correlation; 0.5 ≤ |r| <  0.7 – medium correlation; 0.3 ≤ |r| <  0.5 – low correlation; |r| <  0.3 – very low correlation.

To measure validity, related validity was determined by showing the correlations of variables described in the literature (self-efficacy - GSES, dispositional optimism – LOT-R) with the area assessed with the CLEI-19 scale.

Generalised Self Efficacy Scale (GSES) measures how strongly an individual believes in their ability to manage challenging situations and difficulties. In our study, it contained 10 statements referring to various individual features of character, which the students ticked as true or false for them. The scale ranged from 0 to 40 points. Higher values paralleled with a higher sense of self-efficacy. The Polish version of the scale was developed by R. Schwarzer, M. Jerusalem, and Z. Juczyński and has shown moderately high validity and reliability. Cronbach’s alpha coefficient for the scale amounts to 0.85 [21].

Another test also used in the study was the Life Orientation Test - Revised (LOT-R), created by M. F. Scheier, Ch. S. Carver, and M. W. Bridges. The tool was adapted to Polish conditions by R. Poprawa and Z. Juczyński. The LOT-R tool is used to measure dispositional optimism. It should be noted that, in difficult situations, optimistic people are more likely to apply emotion-based strategies such as acceptance and sense of humour. Optimism is often treated as one of the critical personal resources conditioning a person’s physical, mental, and emotional well-being [21].

### Participants

The study population consisted of 307 students, including 96.1% women (*N* = 295) and 3.9% men (*N* = 12). The average age of the respondents was 20.82 ± 1.53, with their age ranging from 19 (*N* = 49) to 34 (*N* = 1). The average age of the women was 20.78 ± 1.33, while the average age of the men was 21.92 ± 4.06. 20- and 21-year-olds were the largest age groups (*N* = 90, i.e. 29.3%, and *N* = 86, i.e. 28.0%, respectively). 20.2% of the participants were 22 years old (*N* = 62), and a few were slightly older than 22 (*N* = 20, i.e. 6.5%). First-year students constituted 40.1% of all the respondents (*N* = 123). 37.5% of the participants were sophomores (*N* = 115), and 22.5% were third-year students (*N* = 69) (Table 1).

**Table 1:** Characteristics of the study sample

Quality		Values
*Age*	M ± SD	20.82 ± 1.53
	Me	21
	quartiles	20–22
	19 years old	*N* = 49 (16%)
	20 years old	*N* = 90 (29,3%)
	21 years old	*N* = 86 (28%)
	22 years old	*N* = 62 (20,2%)
	> 22 years old	*N* = 20 (6,5%)
*Sex*	Females	*N* = 295 (96.09%)
	Males	*N* = 12 (3.91%)
*Study year*	I	*N* = 123 (40.07%)
	II	*N* = 115 (37.46%)
	III	*N* = 69 (22.48%)

*M* average, *Me* median, *SD* standard deviation, *N* numer of students

## Results

Cronbach’s Alpha for the Clinical Facilitator Support of Learning scale and the Satisfaction with Clinical Placement scale is 0.949 and 0.901, respectively (test). In the re-test Cronbach’s Alpha for the Clinical Facilitator Support of Learning scale is 0.95 and for the Satisfaction with Clinical Placement scale is 0.909. Therefore, the scale is reliable.

All items have positive discriminating power, which means that they correlate positively with other items included in the scale, which is a highly desirable result. Furthermore, excluding any of them has no effect on alpha (Table 2).

**Table 2:** Clinical Learning Inventory items and discriminating power

Clinical Facilitator Support of Learning			Satisfaction with Clinical Placement		
*Item*	*Alpha with the item excluded*	*Discriminating power (item-total correlation)*	*Item*	*Alpha with the item excluded*	*Discriminating power (item-total correlation)*
CLEI1	0.939	0.916	CLEI3	0.89	0.699
CLEI2	0.954	0.372	CLEI5	0.888	0.705
CLEI4	0.943	0.795	CLEI7	0.895	0.635
CLEI6	0.938	0.924	CLEI11	0.877	0.794
CLEI8	0.944	0.802	CLEI13	0.874	0.822
CLEI9	0.944	0.774	CLEI15	0.885	0.747
CLEI10	0.939	0.928	CLEI19	0.896	0.638
CLEI12	0.944	0.809			
CLEI14	0.947	0.675			
CLEI16	0.944	0.767			
CLEI17	0.942	0.828			
CLEI18	0.951	0.51			

The correlation values obtained for both subscales in the test-retest sample at the level of statistical significance with p below 0.001 (Table 3).

**Table 3:** Pearson’s correlation coefficient between subscales in the double test-retest study

Lp	Subscale	Result
1.	*Clinical Facilitator Support of Learning - Clinical Facilitator Support of Learning*	*r* = 0.54 *p* = 0.0001*
2.	*Satisfaction with Clinical Placement – Satisfaction with Clinical Placement*	*r* = 0.48 *p* = 0.001*

*p* level of significance, *r* Perason’s correlation

The average number of points on the Support scale was 40.27 (SD = 10.71), which gives 3.36 points per question. Hence, a conclusion can be drawn that the students did not have any strong opinion as to the teacher’s support. The average number of points on the Satisfaction scale was 15.78 (SD = 5.1), which gives 2.25 points per question. Therefore, a conclusion can be drawn that the students were rather unhappy with their clinical placement (Table 4).

**Table 4:** Correlation between Clinical facilitator support of learning, satisfaction with clinical placement, and the year of nursing studies

CLEI	I year (*N* = 123)	II year (*N* = 115)	III year (*N* = 69)	Kruskal-Wallis test + post-hoc analysis (test Dunna)
*Clinical Facilitator Support of Learning*				
M ± SD	51.86 ± 5.02	32.59 ± 4.91	32.42 ± 4.99	*p* < 0.001 *
Me	52	32	33	
*Satisfaction with Clinical Placement*				
M ± SD	11.46 ± 3.45	18.67 ± 3.59	18.68 ± 4.17	*p* < 0.001 *
Me	11	18	18	

*p* level of significance, *M* average, *Me* median, *SD* standard deviation

To demonstrate content validity, criterion validity was used by showing the correlations of variables described in the literature.

The values of the Spearman’s rank correlation coefficient indicate the existence of a positive correlation between the students’ satisfaction with clinical placement and their life optimism. The more optimistic the students’ attitude towards life in general, the greater their satisfaction with clinical placement. Age correlates significantly and negatively with the perceived teacher support and positively with satisfaction with clinical placement. The older the student, the smaller their perceived level of clinical facilitator support and the greater their satisfaction with their clinical placement. The sense of self-efficacy felt by the students correlates significantly and negatively with their satisfaction with clinical placement; the stronger the students’ sense of self-efficacy, the lower their satisfaction with clinical placement (Table 5).

**Table 5:** Correlation between self-efficacy, age, life optimism of nursing student, and clinical facilitator support and satisfaction with clinical placement

	CLEI	rho Spearmana	***p***	Direction of a relationship
*GSES*	Clinical Facilitator Support of Learning	0.086	*p* = 0.132	–
	Satisfaction with Clinical Placement	−0.123	*p* = 0.031*	negative
*Age*	Clinical Facilitator Support of Learning	0.545	< 0.0001*	positive
	Satisfaction with Clinical Placement	−0.462	< 0.0001*	positive
*LOT-R*	Clinical Facilitator Support of Learning	−0.001	*p* = 0.983	–
	Satisfaction with Clinical Placement	0.139	*p* = 0.001*	positive

*Spearman’s rho* rank correlation, *p* level of significance

## Discussion

The findings of the research provides the validity and reliability of this updated scale for the evaluation of student perceptions of their clinical facilitator’s support of learning and their satisfaction with clinical placement. Results from earlier CLEI studies show that nursing students are engaged in the completion of tasks, but with varying degrees of intensity [8, 22, 23]. However, accounts of students performing monotonous and non-nursing tasks [24] indicate that some placement areas may restrict opportunities to undertake more challenging responsibilities, thereby limiting the possibilities to acquire essential knowledge of critical and clinical judgement [25]. Clinical learning environment can provide unrealistic expectations of nursing and insufficient learning opportunities. That means the quality of the environment is very important in nursing education and therefore, there is a need to monitor the quality and management of that placement. The results of our study indicate that students are quite satisfied with their clinical learning environment and the results obtained by other authors are consistent with our study [6].

The level of student satisfaction with their clinical environment fluctuates and the overall score is vulnerable due to several variables such as the intensity of engagement, affiliation to a team, and involvement in engaging undertakings as well as quality of the environment [10, 26, 27].

Chan and Ip viewed satisfaction as an educational effect demanding the consideration of nursing specialists and legislators [28]. However, two Iranian studies showed that it was not being taken into account in clinical educational environments [29, 30].

The authors’ study indicated that the students were rather dissatisfied with their clinical placement. The study by Ramsbotham et al. [31], on the other hand, suggests that students were somewhat satisfied with their placement. In studies by Salamonson et al. [32] as well as Antohe et al. [33], research students were satisfied with their clinical placement.

Student perceptions of the clinical learning environment in our research varied significantly between both groups and respondents depending on their years of studies. Similar results were seen by Ramsbotham et al. [31].

The analysis demonstrates that the first-year students noticed significantly greater teacher support but were considerably less satisfied with their clinical placement than the second- and third-year students. While similar results were obtained by other authors [12, 31, 34], conflicting results were obtained by Pitkänen et al. [6]; their study revealed third-year healthcare students as the most dissatisfied with their clinical learning environment and instructional support while undertaking their clinical placement.

The values of the Spearman’s rank correlation coefficient indicate that there is a positive correlation between the students’ satisfaction with clinical placement and their life optimism. The more optimistic the students’ attitude towards life in general, the greater their satisfaction with their clinical placement. The results of a study published by Fang et al. [35] showed that, among undergraduate nursing students, the educational environment was strongly associated with career motivation, while optimism was only weakly connected.

Age correlates both significantly and negatively with perceived teacher support (rho = − 0.462, *p* < 0.0001) and positively with satisfaction with clinical placement (rho = 0.545, *p* < 0.0001). The older the student, the smaller their perceived clinical facilitator support and the greater their satisfaction with clinical placement. Research conducted by D’Souza et al. [36] indicated that a more mature age and graduation from several clinical courses were crucial in the perceived satisfaction with the clinical learning environment.

The analysis has shown that the level of self-efficacy could predict satisfaction with clinical experience. The sense of self-efficacy felt by the students correlates significantly and negatively with their satisfaction with clinical placement (rho = − 0.123, *p* = 0.031). The stronger the students’ sense of self-efficacy, the lower their satisfaction with clinical placement. Similar outcomes have been achieved by other authors [37] and, in addition, Flott and Linden [5] emphasised that CLE experiences should be measured over time, evaluating the influence on student self-confidence. Self-efficacy is a critical outcome in nursing education since high levels are associated with finding it more comfortable to transform from a student to a nursing professional [38].

CLEI-19 is a tool that provides an insight into the level of nursing student satisfaction with two areas of the learning environment, shared by most frameworks of clinical education. It could, therefore, be a valuable instrument for assessing the quality of clinical learning in Polish conditions. The inventory may give feedback while designing and implementing clinical aspects of learning in nursing education and a useful strategy for improving the quality of courses. It could also be a tool to guide educators in their work, as previous results of research have shown [39, 40].

Mahasneh et al. emphasised the huge need for a preparation programme for newly employed supervisors and training with identifying their competency level [41].

Nursing student’s perspective in the clinical placement is an important component of educational planning [11]. It seems essential for future research on the quality of clinical education in Poland that decision makers have a better understanding of student perspectives on the matter.

### Study limitation

This study was conducted in only one Medical University; therefore, the results might not be readily generalised to other nursing institutions across Poland. The study sample was dominated by female students, which could potentially cause bias. Further research is needed also to explore the impact of other factors on nursing student’s perception of their clinical learning environment and also should be undertaken at multiple universities.

## Conclusions

1. The CLEI–19 Scale used in the study generally shows acceptable criterion validity and reliability. The reliability of the test in the Polish version is consistent with the reliability obtained in the original author’s validation. It could, therefore, be a valuable instrument to assess the quality of clinical learning in Polish conditions.
2. First-year students noticed significantly greater teacher support but are considerably less content with their clinical placement than the second- and third-year students. The older the student, the smaller their perceived clinical facilitator support and the greater their contentment with clinical placement.
3. The more optimistic the students’ attitude towards life in general, the greater their contentment with clinical placement. The stronger the students’ sense of self-efficacy, the lower their satisfaction with clinical placement.

## Data Availability

The datasets used and analysed during the current study are available from the author based on reasonable request.

## Abbreviations

CLEI: Clinical Learning Environment Inventory
GSES: Generalised Self Efficacy Scale
LOT-R: Life Orientation Test - Revised
